# Insecticidal Properties of Capsaicinoids and Glucosinolates Extracted from *Capsicum chinense* and *Tropaeolum tuberosum*

**DOI:** 10.3390/insects10050132

**Published:** 2019-05-06

**Authors:** José L. Claros Cuadrado, Elder O. Pinillos, Richard Tito, Carlos Seguil Mirones, Norma N. Gamarra Mendoza

**Affiliations:** 1 Faculty of Forest and Environmet Science, National University of Central Peru, Huancayo 12000, Peru; jclaroscuadrado@gmail.com; 2 National Institute of Agricultural Innovation—Experimental Station of Santa, Huancayo 12000, Peru; ofeliapinillos@hotmail.com; 3 School of Biology, National University of San Antonio Abad in Cusco, Cusco 08001, Peru; 4 Department of Biology, University of Miami, Coral Gables, FL 33146, USA; 5 High Mountain Research Center National University of Central Peru, Huancayo 1200, Peru; 6 Faculty of Food Industry Engineering, National University of Central Peru, Huancayo 12000, Peru; cseguil60@yahoo.com

**Keywords:** aphid pests, bioinsecticide, climate change, eco-friendly products, natural insecticides, natural products, pest control

## Abstract

Food security and biodiversity conservation are threatened by the emergence and spread of pest and pathogens, and thus there is a current need to develop pest management strategies that are sustainable and friendly to the environment and human health. Here, we performed laboratory and field bioassays to evaluate the insecticidal effects of several concentrations of capsaicinoids and glucosinolates (separately and mixed) on an aphid pest (*Aphis cytisorum*). The capsaicinoids were extracted from the fruits of *Capsicum chinense* and glucosinolates from the tubers of native Andean crop *Tropaeolum tuberosum*. We found that both capsaicinoids and glucosinolates have a biocidal effect on *A. cytisorum*, acting within a fairly short time. Under laboratory conditions, the toxicity of the compounds increased in relation to their concentrations, causing a high percentage of mortality (83–99%) when the aphids were exposed to dilutions of 10% capsaicinoids, 75–100% glucosinolates, or a mixture of 10% capsaicinoids and 90% glucosinolates. The mortality of aphids sprayed in the field with 5% capsaicinoids, 50% glucosinolates, or with a mixture of 5% capsaicinoids and 45% glucosinolates reached 87–97%. Results obtained from laboratory and field experiments were consistent. Our results suggest the potential use of bioinsecticides based on capsaicinoids and/or glucosinolates as an effective alternative to synthetic pesticides.

## 1. Introduction

The predicted increase in the abundance and distribution of pests and pathogens in response to climate change threatens to cause a severe impact on both wild and agricultural plant species [1,2]. For instance, a recent field experimental study showed that pests and diseases will be one of the main causes of crop production loss under future climate change conditions [3]. Chemicals are commonly used to counteract the impact of phytophagous, mainly on crops. In fact, the use of pesticides has increased in recent decades, and this rising trend will likely accelerate in the coming decades [4,5]. However, it is widely known that synthetic pesticides affect non-target organisms as well as the environment e.g., [6]. In addition, the inappropriate use of chemicals facilitates the acquisition of pest resistance to common chemicals [4], which in turn leads to an increase in the use of even more potent pesticides. Therefore, proper pest control management should be prioritized to maintain a reasonably healthy condition for human health and the environment [7]. There are several pieces of evidence that show that the use of plants with insecticidal properties is one of the main ecologically friendly and economically feasible alternatives to synthetic pesticides [8,9].

Previous studies showed evidence that capsaicinoids and glucosinolates have biocidal activity against phytophagous insects [10,11]. Glucosinolates are amino acid-derived natural plant compounds that act as a defense against herbivores. They are found in several plant species, including in the genus *Tropaeolum* [12,13]. Under natural conditions, glucosinolates can be hydrolyzed by myrosinases to (primarily) benzyl isothiocyanates upon tissue damage [14]. On the other hand, capsaicinoids are a group of secondary metabolites found only in the genus *Capsicum* [15]. Among the capsaicinoid components, capsaicin and dihydrocapsaicin are responsible for approximately 90% of the total pungency [16,17].

Aphids are one of the largest and most important pests in wild and agricultural plant species [18], and their population is expected to increase due to climate change—elevated temperatures accelerate aphid reproduction and increase their fecundity [19,20]. Aphids are specialized insects that feed on the phloem sap of vascular plants. Therefore, aphids have a direct impact on the host plant fitness, mainly due to the loss of sugars and defense compounds (secondary metabolites) that are abundant resources in the sap [21,22]. Aphids are also disease vectors—due to their feeding mechanism (i.e., sap-sucking herbivore), they can transmit pathogens to the interior of the host plant tissue [23,24]. In addition, the intestinal tract of aphids has evolved to exude the excess of sugars consumed in the form of honeydew, a sugary substance that facilitates the development of pathogens such as sooty mold fungus, which can cover the surface of the plant and alter its photosynthetic and respiratory processes [25]. These indirect effects often become more significant to the survival of the plant than the direct effects caused by aphids [22,26].

In the Andes, one of the most biodiverse ecosystems in the world [27], the invasive exotic plant *Spartium junceum* L. (Fabaceae, commonly called Spanish broom or retama) has been massively infested by *Aphis cytisorum* Hartig (Homoptera: Aphididae) since 2005 [26]. As expected, the infestation of aphids facilitated the rapid proliferation of sooty mold fungus. This aphid–fungus damage led to the high mortality of *S. junceum* populations [26]. Despite that, in the Andes, *A. cytisorum* is currently reported to infest only *S. junceum* [26,28], but there is a risk that this polyphagous aphid [29,30,31] will infest other Andean plant species (agricultural as well as wild plants) [26]. Therefore, there is a real need to carry out adequate control management to avoid the possible ecological and economic impact of this and other pests.

Here, we evaluated the potential bioinsecticidal activity of glucosinolates and capsaicinoids on *A. cytisorum*. We performed laboratory and field experiments to test the toxicity of different concentrations of glucosinolates and capsaicinoids, separately or mixed. The results of this study can help to promote the use of natural compounds with biocidal effects for pest management, thus reducing the risks of chemical pollution. The glucosinolates were extracted from tubers of the Andean plant *Tropaeolum tuberosum* (Tropaeolaceae, locally known as mashua or isaño) and the capsaicinoids from the placentas of the fruits of *Capsicum chinense* (Solanaceae, locally called as ají panca). *Tropaeolum tuberosum* is cultivated throughout the Andes (from Colombia to Argentina) between 2100 and 4000 m of elevation, and the Andes of Peru are the largest producing region [32,33]. On the other hand, *C. chinense* is cultivated in tropical ecosystems from sea level to the high elevations of the Andes [34]. These two species are widely produced and used as food and in the food-processing industry. In the case of *C. chinense*, consumers prefer the pericarp of the fruit and the placenta is discarded [35], and this resource was used in this study to extract the capsaicinoids.

## 2. Materials and Methods

### 2.1. Compound Extraction and Quantification

The fruits of *Capsicum chinense* were obtained from the local chili industry (Sazon Lopesa, Peru). The fruits were produced on the Peruvian coast (Tacna; 18°00′52″ S, 70°15′13″ W) during the 2013/2014 sowing period. On the other hand, tubers of *Tropaeolum tuberosum* were obtained from the farmers of the Pampas district of the Huancavelica region, Peru (12°23′53″ S; 74°52′04″ W) and grown during the 2014/2015 sowing period. Both species were grown free of synthetic agrochemicals. In the case of *C. chinense* fruits, the placentas and interlocular septa (the structure that divides the internal cavity of the fruit) were used, since these structures contain approximately 5–10 times more capsaicinoids in comparison to the pericarp and seeds [35,36]. The capsaicinoids are secondary metabolites derived from benzylamides [36,37].

The extraction of glucosinolates and capsaicinoids was performed in the food chemistry laboratory at the Universidad Nacional del Centro del Perú. Each sample of tubers of *T. tuberosum* and placentas of *C. chinense* were dried at 45 °C until its moisture content reached 10–12%. In the case of *T. tuberosum*, prior to drying, the tubers were immersed into hot water (70 °C) for five minutes in order to inhibit the myrosinase enzymes and thus avoid hydrolysis. Dry samples were crushed and sieved in order to obtain uniform particle sizes (0.43–1.0 mm). These particles were pretreated under supercritical CO_2_ at a pressure of 300 bar at 60 °C and 400 bar at 40 °C for *T. tuberosum* and *C. chinense*, respectively. This procedure was carried out to improve the extraction of the focal compounds. Then, the glucosinolates were obtained from crushed and pretreated samples of tubers of *T. tuberosum* using 70% methanol at 75 °C and agitating constantly for 20 minutes [38]. The mixture was filtered, centrifuged at 4500 rpm for 15 min, and then stored at 4 °C. On the other hand, the capsaicinoids were extracted from crushed and pretreated samples of placentas of *C. chinense* using absolute ethanol at 60 °C and stirring at 270 rpm for 12 h, following the procedures commonly used in the literature [39,40]. The extracted compounds were used to perform the toxicity tests on aphids in the laboratory and in the field.

The capsaicinoids and glucosinolates were quantified by high-performance liquid chromatography (HPLC) using an ultra-fast liquid chromatograph (UFLC) system(Shimadzu, Tokyo, Japan) with a pinnacle II column (C18, 250 mm, 5 µm x 4.6 mm). The capsaicinoids were separated at 30 °C and glucosinolates at 28 °C with a flow speed of the mobile phase of 1.5 mL min^−1^ and 0.8 mLmin^−1^, respectively. The mobile phase for capsaicinoids was prepared with water at 1% of acetic acid and acetonitrile (50:50 v/v), while for glucosinolates with 0.01% trifluoroacetic acid and methanol (20:80 v/v). The elution of capsaicinoids was performed under isocratic conditions for 20 min and the elution of glucosinolates under gradient conditions for 40 min. Capsaicinoids were recorded at a 280 nm wavelength and glucosinolates at 229 nm. The calibration curves were previously elaborated using capsaicinoid (capsaicin, dihydrocapsaicin, and nordihydrocapsaicin) and glucosinolate (sinigrin, glucotropaeoline, and gluconasturtine) standards.

### 2.2. Laboratory and Field Bioassays

The sampling of aphids and field experiments were carried out in the central Peruvian Andes (Mantaro Valley, 12°15′46″ S, 73°58′44″ W) in May 2017. For the first laboratory experiment, a total of 1000 aphids were randomly collected from 10 adult plants of *S. junceum*. Immediately after collection, the insects were carefully transported (in polyethylene containers) to the laboratory (Integrated Pest Management Laboratory at the Instituto Nacional de Innovación Agraria in Peru) to perform the toxicity tests on the same day that they were collected. To this end, capsaicinoid and glucosinolate extracts were diluted using distilled water to obtain four different concentrations of each compound and a mixture of the two compounds (mg/10 mL). Specifically, the concentrations (v/v) were capsaicinoids at 1%, 2.5%, 5%, and 10%; glucosinolates at 25%, 50%, 75%, and 100%; and a mixture of capsaicinoids and glucosinolates in proportions of 1:24%, 2.5: 47.5%, 5:70%, and 10:90%. In order to test insecticidal activity, 50 adults of *A. cytisorum* were exposed to each of these bioinsecticide dilutions in Petri dishes (10 cm in diameter and 15 mm deep). Each treatment was replicated three times. The bioinsecticides were administered only once, by spraying the dilutions equivalent to 2 mL per Petri dish. The number of dead insects in each Petri dish was recorded 30 minutes after spraying.

**Figure 1:**
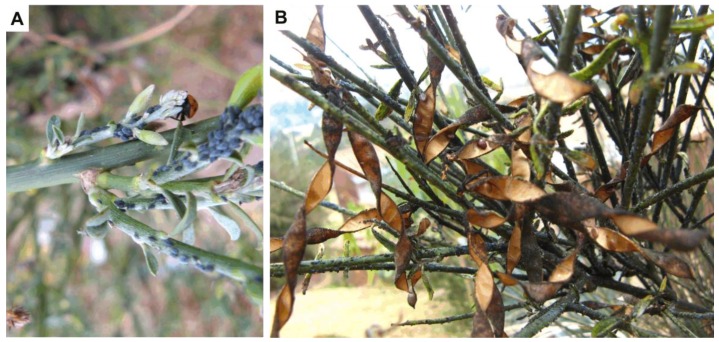
Images of *Spartium junceum* L. infested by *Aphis cytisorum* in the Peruvian Andes. (**A**) Apical branches, the tender region of the branch where a massive infestation of aphids can be observed. (**B**) Adult plant of *S. junceum* infested by *A. cytisorum* and sooty mold fungus *Capnodium* sp. (black powder that has blackened the green stems of the plant). Photos by Richard Tito.

The second experiment was conducted in the field. Nine adult plants of *S. junceum* (~2 m height and ~1.2 m canopy diameter) infested by *A. cytisorum* were randomly chosen, >5 m apart from each other. The total number of alive aphids in the apical part of 12 randomly selected branches per plant was recorded. The apical part of the branches is where aphids tend to agglomerate (Figure 1). In three of these 12 randomly selected branches (where the aphids were quantified), one of the following four treatments was sprayed: dilution of (1) 5% capsaicinoids, (2) 50% glucosinolates, (3) mixture of 5% capsaicinoids and 45% glucosinolates, and (4) distilled water (control). In other words, the four treatments were sprayed three times (i.e., on three branches) on each plant. Compound concentrations were chosen based on the results from our first laboratory experiment. The administration of bioinsecticides was carried out in the morning (7 am), using a sprinkler that allowed homogeneous spray. After 24 and 168 h of administration, the number of dead aphids was recorded (i.e., field experimentation lasted seven days). At the time of setting up the experiment, a white blanket was spread at the base of the plant to check the dead aphids that fell off the branches.

### 2.3. Statistical Analysis

The effect of the different concentrations of the bioinsecticides on the mortality of *A. cytisorum* was tested using analysis of variance (ANOVA): Two-way ANOVA was used for the results from the laboratory experiments and repeated-measures ANOVA in the case of field experiments. The Tukey test was used to conduct multiple comparisons between treatments. The assumptions for performing parametric analyses were verified by means of residue exploration.

## 3. Results

### 3.1. Content of Capsaicinoids and Glucosinolates

The total concentration of capsaicinoids (capsaicin, dihydrocapsaicin, and nordihydrocapsaicin) in the extracts of the placentas of *Capsicum chinense* was 1.48 mg.mL^−1^, and the concentration of glucosinolates (gluconasturtin, glucotropaoelin, and glucoaubrietin) in the extracts of tubers of *Tropaeolum tuberosum* was 1.62 mg.mL^−1^.

### 3.2. Toxicity under Laboratory Conditions

**Figure 2:**
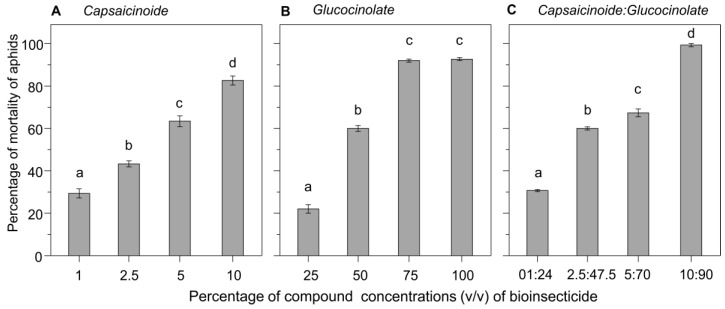
Percentage of mortality of *Aphis cytisorum* exposed (in the laboratory) to different concentrations of (**A**) capsaicinoids, (**B**) glucosinolates, and (**C**) a mixture of these two compounds. Different letters above error bars indicate significant statistical difference (*P* < 0.05) in mean values between treatments.

The level of toxicity of the bioinsecticides sprayed on *Aphis cytisorum* depended on the compounds (i.e., capsaicinoids, glucosinolates, or the mixture of these two compounds) and their concentrations (*F*_2.6_ = 61.6; *P* < 0.001). When aphids were sprayed with dilutions of capsaicinoids, aphid mortality increased in direct proportion to the increase in concentration (*F*_3.8_ = 215.9; *P* < 0.001; Figure 2A). Aphids sprayed with 10% capsaicinoids experienced high percentages of mortality (~82%; Figure 2A). The toxicity of glucosinolates also varied according to the concentration, with higher lethality at higher concentrations (*F*_3,8_ = 1095.5; *P* < 0.001; Figure 2B). Glucosinolates at 75% and 100% caused a high and similar percentage of mortality (~92%) of aphids (Figure 2B). The mixture of capsaicinoids and glucosinolates significantly increased the toxicity effects compared to the activity of each compound separately (*F*_3,8_ = 1067.8; *P* < 0.001; Figure 2C), and toxicity also increased with compound concentration (Figure 2C). The mixture of 10% capsaicinoids and 90% glucosinolates caused the highest percentage of mortality (99%) of aphids (Figure 2C).

### 3.3. Toxicity under Field Conditions

**Figure 3:**
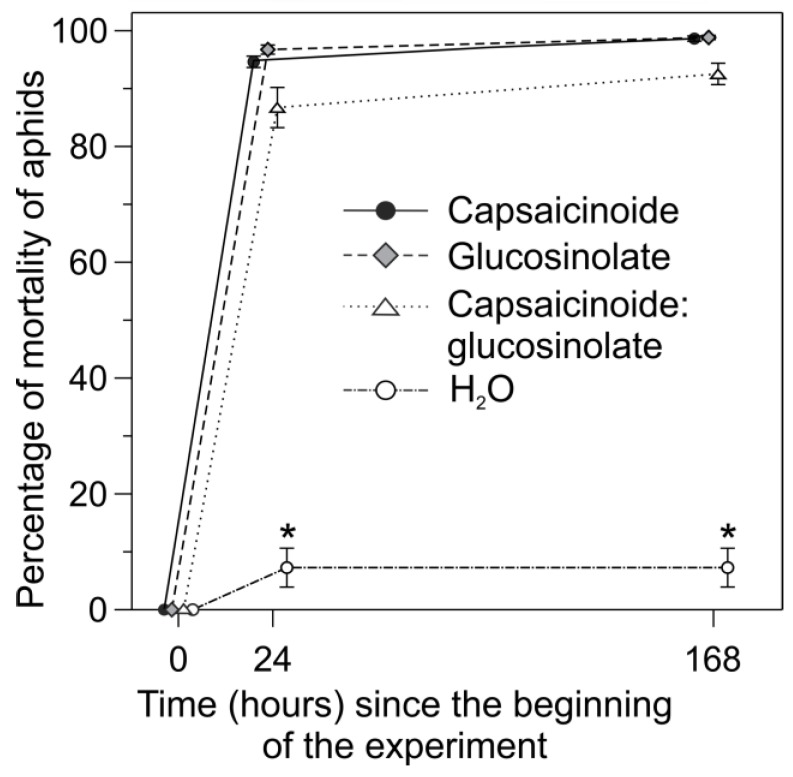
Percentage of mortality of *Aphis cytisorum* exposed (in the field) to dilutions of capsaicinoids at 5%, glucosinolates at 50%, and a mixture of 5% capsaicinoids and 45% glucosinolates. The results are shown for a period of 168 h after the aphids were sprayed with the bioinsecticides. Asterisks above error bars indicate significant difference (*P* < 0.05) between treatments.

All treatments (i.e., 5% capsaicinoids, 50% glucosinolates, and a mixture of 5% capsaicinoids and 45% glucosinolates) caused a high percentage of mortality of aphids (F_3,32_ = 22.6; *P* <0.001; Figure 3). When the aphids were sprayed with distilled water, however, mortality was insignificant (Figure 3), suggesting that the manipulation in our study system did not influence the results. In all treatments, a high mortality of aphids (87–97%) was recorded within 24 h after spraying with bioinsecticides (Figure 3).

## 4. Discussion

Consistent results found in the laboratory and the field experiments strongly suggest that capsaicinoids and glucosinolates can be an ecofriendly alternative to synthetic pesticides. Our results clearly reveal that dilutions of capsaicinoids (from 5% in concentration) and glucosinolates (from 50% in concentration) can effectively control *Aphis cytisorum*. In addition, the rapid biocidal activity of compounds (within 30 minutes) is important for application in the field, where some abiotic factors (e.g., rain) can influence the effectiveness of pesticides. *Aphis cytisorum* has been reported as an aggressive pest of *Spartium junceum* in the Andes and threatens other Andean plant species [26], which could lead to serious ecological and economic consequences. In other regions, *A. cytisorum* is a pest that affects several plant species [30,31]. Our results are also consistent with results from several previous studies that have shown clear evidence that capsaicinoids and glucosinolates are efficient bioinsecticides for controlling several other species of herbivores. The capsaicin extracted from *Capsicum annum* (a species related to *C. chinese*) caused the mortality of approximately 97% of *A. myzus* [37]. The toxicity of capsaicin dilutions has been previously verified on potato beetle larvae (*Leptinotarsa decemlineata*) [41] and on *Tenebrio molitor* [11]. Similarly, glucosinolates have been shown to be an effective bioinsecticide against aphids [42] and larvae that attack corn root (*Diabrotica virgifera*) and grain (*Oryzeaphilus surinamensis*, *Tribolium castaneum*, *Rhyzopertha domínica*) [10,43]. Furthermore, it was shown that glucosinolates are toxic to *Aedes aegypti* larvae [10], a mosquito that transmits multiple diseases (dengue, Chikungunya, and zika) that affect millions of people [44,45].

As expected, the mixture of capsaicinoids and glucosinolates increased the biocidal effect of these compounds in laboratory conditions, although that effect was not as obvious in the field experiments. Previous studies that tested the effect of a mixture of two bioinsecticides also showed a significant increase in biocidal activity. For example, Edelson et al. [46] showed that capsaicinoids administered as the only biocidal component caused low percentages of mortality of *A. persicae*, but acting simultaneously with other insecticides, generated a synergistic effect that caused higher levels of mortality. Likewise, Olszewska et al. [11] showed that capsaicin intensifies the effect of pyrethroids, since their simultaneous administration increased the metabolic rate of *Tenebrio molitor* larvae and caused intoxication. Therefore, a mixture of bioinsecticides can increase effectiveness, but if this is not feasible, our results indicate that the application of capsaicinoids or glucosinolates alone is also highly effective.

The concentrations of capsaicinoids and glucosinolates found in the placentas of *C. chinense* and tubers of *T. tuberosum* were relatively high, indicating that these plant structures are valuable resources for the extraction of these compounds. Nevertheless, it is important to note that the content of capsaicinoids in *C. chinense* fruits and glucosinolates in *T. tuberosum* tubers varies depending on the varieties and several other abiotic factors, such as temperature, humidity, and soil properties [15,38,47]. On the other hand, the placentas of the fruits of *C. chinense* that are commonly discarded would be low cost raw materials [35].

## 5. Conclusions

Our results strongly suggest that bioinsecticides (particularly capsaicinoids and glucosinolates) can be used for pest management. This may represent a viable, effective, and ecofriendly alternative to synthetic pesticides. In addition, the use of raw material discarded in industry or gastronomy (such as the placentas of *C. chinense*) is essential to achieve this goal, considering that the land and climatic conditions required for agricultural production to meet the growing food demand are also threatened [3,48].
